# FRAP Analysis on Red Alga Reveals the Fluorescence Recovery Is Ascribed to Intrinsic Photoprocesses of Phycobilisomes than Large-Scale Diffusion

**DOI:** 10.1371/journal.pone.0005295

**Published:** 2009-04-20

**Authors:** Lu-Ning Liu, Thijs J. Aartsma, Jean-Claude Thomas, Bai-Cheng Zhou, Yu-Zhong Zhang

**Affiliations:** 1 State Key Lab of Microbial Technology, Marine Biotechnology Research Center, Shandong University, Jinan, People's Republic of China; 2 Department of Biophysics, Huygens Laboratory, Leiden University, Leiden, The Netherlands; 3 UMR 8186 CNRS & Ecole Normale Supérieure, Biologie Moléculaire des Organismes Photosynthétiques, Paris, France; Geroge Mason University, United States of America

## Abstract

**Background:**

Phycobilisomes (PBsomes) are the extrinsic antenna complexes upon the photosynthetic membranes in red algae and most cyanobacteria. The PBsomes in the cyanobacteria has been proposed to present high lateral mobility on the thylakoid membrane surface. In contrast, direct measurement of PBsome motility in red algae has been lacking so far.

**Methodology/Principal Findings:**

In this work, we investigated the dynamics of PBsomes in the unicellular red alga *Porphyridium cruentum in vivo* and *in vitro*, using fluorescence recovery after photobleaching (FRAP). We found that part of the fluorescence recovery could be detected in both partially- and wholly-bleached wild-type and mutant F11 (UTEX 637) cells. Such partial fluorescence recovery was also observed in glutaraldehyde-treated and betaine-treated cells in which PBsome diffusion should be restricted by cross-linking effect, as well as in isolated PBsomes immobilized on the glass slide.

**Conclusions/Significance:**

On the basis of our previous structural results showing the PBsome crowding on the native photosynthetic membrane as well as the present FRAP data, we concluded that the fluorescence recovery observed during FRAP experiment in red algae is mainly ascribed to the intrinsic photoprocesses of the bleached PBsomes *in situ*, rather than the rapid diffusion of PBsomes on thylakoid membranes *in vivo*. Furthermore, direct observations of the fluorescence dynamics of phycoerythrins using FRAP demonstrated the energetic decoupling of phycoerythrins in PBsomes against strong excitation light *in vivo*, which is proposed as a photoprotective mechanism in red algae attributed by the PBsomes in response to excess light energy.

## Introduction

Photosynthetic organisms are able to capture solar energy using light-harvesting antennae in the photosynthesis process [1], [2]. In the eukaryotic red algae and most of the prokaryotic cyanobacteria, the light-harvesting antennae are extrinsic supramolecular complexes, designated phycobilisomes (PBsomes). PBsomes are arranged on the surface of photosynthetic membranes [3]–[5], absorbing light and transferring it to the reaction center with high efficiency [6]–[8].

The major components of PBsomes are the phycobiliproteins (PBPs). According to the different properties, PBPs are commonly divided into four main classes: allophycocyanin (APC), phycocyanin (PC), phycoerythrin (PE) and phycoerythrocyanin (PEC) [1], [3], [9]. In the presence of linker polypeptides, PBPs are assembled into two subcomplexes: the core that combines with the membrane-bound photosynthetic reaction centers, and the peripheral rods that attach to the core [7], [10]–[12]. The former is mainly composed of APCs, and the latter contain PCs, PCs+PEs or PCs+PECs, depending on the species [1], [3], [4]. In the unicellular red alga *Porphyridium (P.) cruentum*, the main components of PBPs are PEs, which consist of B-PE and b-PE [13], [14]. These two types of PEs are both heterooligomers, but they differ by the presence/absence of the γ subunit [13], [15], [16].

Fluorescence recovery after photobleaching (FRAP) has been exploited to study the protein diffusion since 1970s [17]–[19]. It has been applied in exploring the mobility of PBsomes in cyanobacteria, suggesting that PBsomes could diffuse laterally on the surface of thylakoid membrane [20]–[22]. Such rapid mobility of PBsomes was further proposed to be a prerequisite for the light-state transition [23], a physiological mechanism that adapts to distribute the excitation light between photosystem I and photosystem II [24]–[26]. To date, the measurement of PBsome mobility in red algae has been lacking. Structural explorations have revealed larger size of the hemiellipsoidal PBsomes in *P. cruentum* compared to the hemidiscoidal PBsomes in cyanobacteria, and significant macromolecular crowding of PBsomes on the thylakoid surface in red algae [14], [27], [28]. These findings raise the question: the rapid and long-range diffusion of PBsomes in red algae may be highly restricted by a combination of macromolecular crowding of membrane surface and specific protein-protein interactions upon the thylakoid surface [29], [30].

In this paper, using FRAP we directly investigated the dynamics of PBsomes in *P. cruentum* wild-type (WT) and mutant F11 cells, as well as the isolated PBsomes *in vitro*. Our findings revealed that the fluorescence recovery observed is due to the intrinsic photoprocesses of PBsomes *in situ*, rather than large-scale PBsome diffusion *in vivo*. Furthermore, *in situ* light-induced energetic decoupling of PBsomes investigated by FRAP was proposed as an energy dissipation mechanism *in vivo* in response to excess light energy.

## Results

### Fluorescence Recovery of Partially Photobleached WT Cell

**Figure 1:**
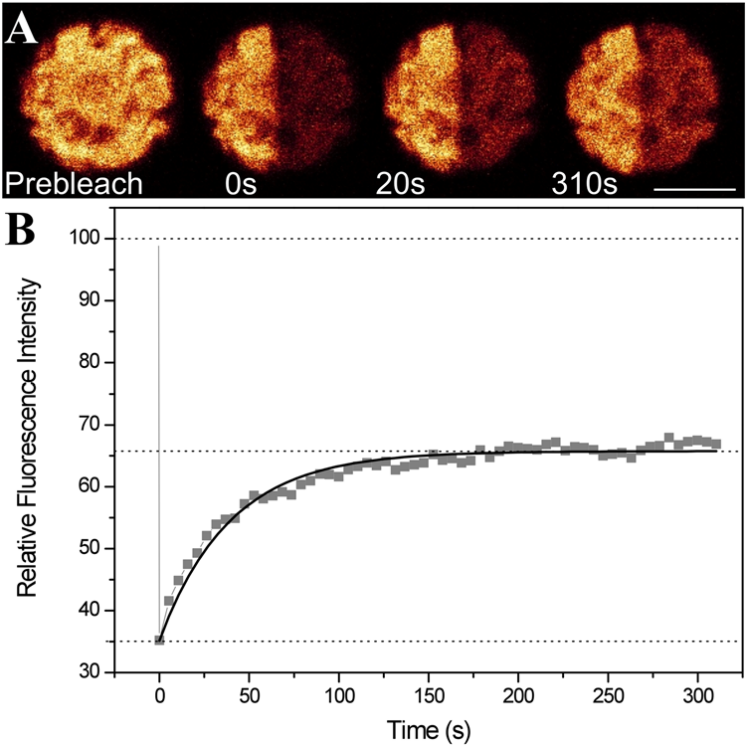
Qualitative FRAP experiments of red alga *P. cruentum* WT cell. A, selected fluorescence images from typical sequences recorded before bleaching, immediately after bleaching of PBsomes, and at various time lapses. Excitation is at 568 nm and detection range is from 650 to 750 nm. Scale bar: 5 µm; B, total fluorescence intensity of bleached cell region as a function of time. The recovery of the fluorescence is presented as square spots and fitted to an exponential function (solid line).

FRAP has been performed to study the mobility of PBsomes on cyanobacterial thylakoid membrane [20], [31]–[33]. Here we applied the same strategy to individual cells of the red alga *P. cruentum* for analyzing the diffusion dynamics of PBsomes upon the thylakoid membranes. In the experiments, confocal fluorescence images were acquired of a particular plane in the cell. We excited the PBsomes in *P. cruentum* cells with a 568 nm laser which was mostly absorbed by PEs in the peripheral PBsome rods. The fluorescence emission of PBsome terminal emitters was detected in the range of 650–750 nm. For pre- and post-scanning 5% of the laser power was applied, and photobleaching was performed by zooming-in scan at 100% laser power (10 mW). Figure 1A shows a typical FRAP image sequence of PBsome emission in a single *P. cruentum* cell. Part of the cellular area was bleached to a depth of 65% and the fluorescence recovery of the bleached area was detected by a post-scan. The recovery of the fluorescence in bleached cellular region is depicted in Figure 1B (dashed line) and adequately fitted to an exponential function (Equation 1, see Materials and Methods). The fluorescence recovery levels off at 66% of the initial fluorescence intensity only (from 35% after bleaching). The recovery rate (*R*) was calculated to be 39.5 s^−1^ with Equation 1. Furthermore, we found that more intense illumination could induce less fluorescence recovery. When the photobleaching reaches 80–90% depth, the fluorescence recovery of PBsomes was much less, though still visible. This was corroborated with previous finding in cyanobacterium *Thermosynechococcus elongates* [33].

### Fluorescence Recovery of Wholly Photobleached WT Cell

**Figure 2:**
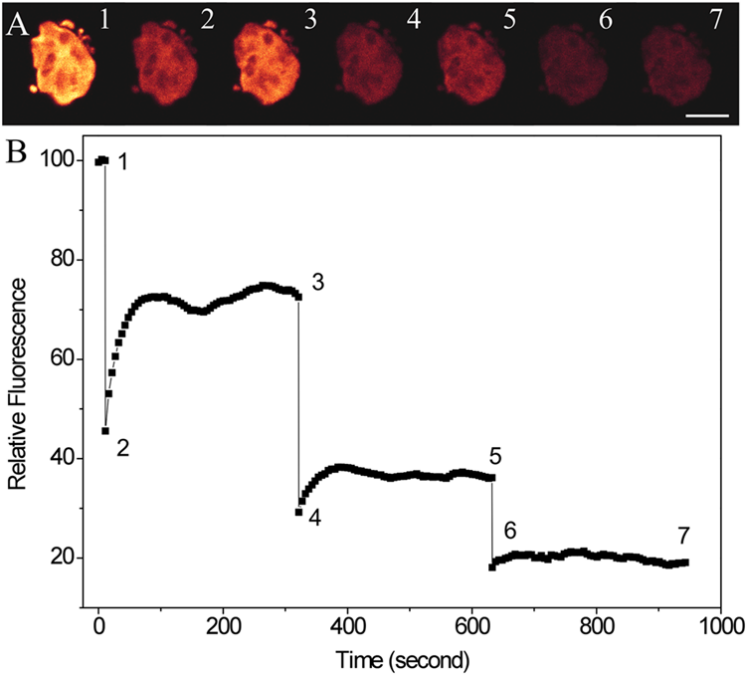
FRAP selected fluorescence images of the *P. cruentum* WT cells. A, wholly bleaching on the cells. Excitation is at 568 nm and detection range is from 650 to 750 nm. Selected fluorescence images from sequences recorded before bleaching, immediately after bleaching of PBsomes, and at various time lapses. Cycle 1: 1-2-3; cycle 2: 3-4-5; cycle 3: 5-6-7; Scale bar: 5 µm; B, one-dimensional bleaching profiles derived from the sequences of fluorescence images of panel A.

To further survey the photodynamics of PBsome complexes, we explored a series of comparative experiments with the aforementioned FRAP procedure to whole cells. Figure 2 shows the time-course photobleaching of the whole *P. cruentum* cell. Three continuous cycles of photobleaching were carried out on the same cell. All the PBsomes were generally bleached by whole-cell scanning with intense laser power (100%). Generally, no fluorescence recovery by the lateral mobility of PBsomes was expected in this case. However, the partial recovery of fluorescent intensity from the whole cell was still observed. It was also found that stronger bleaching leads to less recovery, which has been observed in partially photobleached cell. There is one probability that such fluorescence recovery may be ascribed to the diffusion of PBsomes from neighboring lamellae surfaces. However, we found the fact that scanning with full power caused bleaching of PBsomes not only in the focusing plane, but also perpendicularly, in a few-micrometer flanking areas through the cell (data not shown).

### Fluorescence Recovery of Glutaraldehyde/Betaine-Treated WT Cell

**Figure 3:**
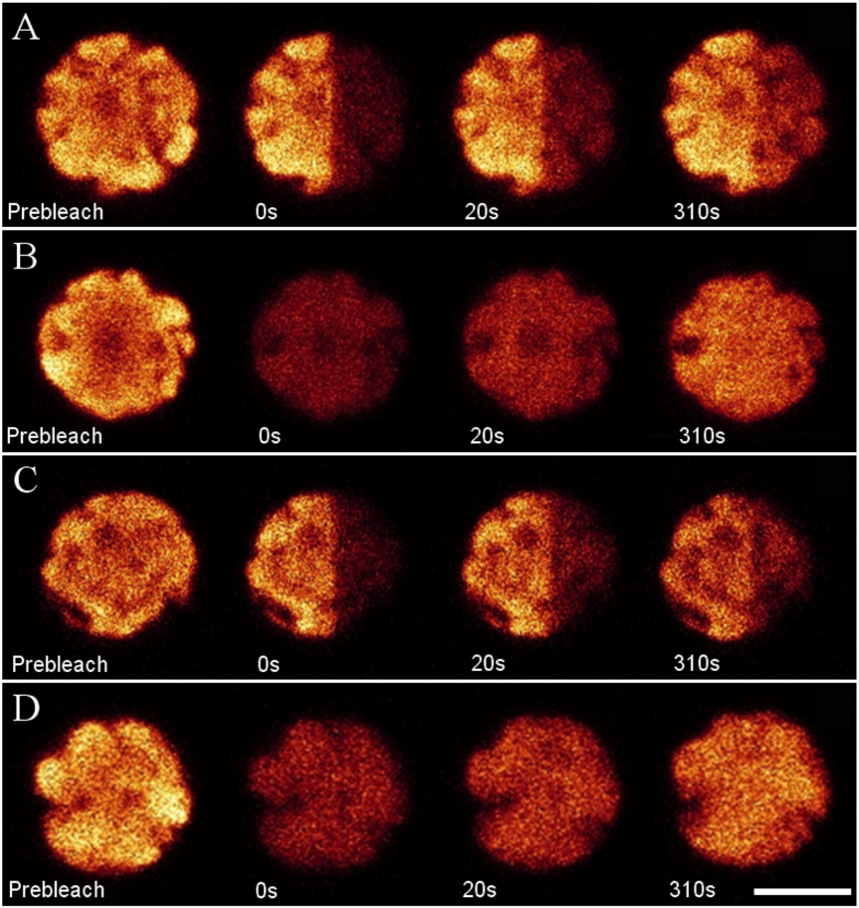
FRAP selected fluorescence images of *P. cruentum* WT cells pretreated with glutaraldehyde/betaine. Excitation is at 568 nm and detection range is from 650 to 750 nm. A, partially-bleached cell pretreated with betaine; B, wholly-bleached cell pretreated with betaine; C, partially-bleached cell pretreated with glutaraldehyde; D, wholly-bleached cell pretreated with glutaraldehyde. Scale bar: 5 µm.

Furthermore, FRAP experiments were performed on whole cells pre-treated with 1% glutaraldehyde or 0.5 M betaine, which have been documented to be able to chemically promote cross-linking between PBsomes and thylakoid membrane [27], [34]–[39]. Figure 3 shows that the fluorescence recovery was unexpectedly detected in all cases. The recovery rates in all conditions were calculated to be constantly 40.0±1.0 s^−1^, similar to the results of the native cells mentioned above.

### Analysis of The Fluorescence Profiles

**Figure 4:**
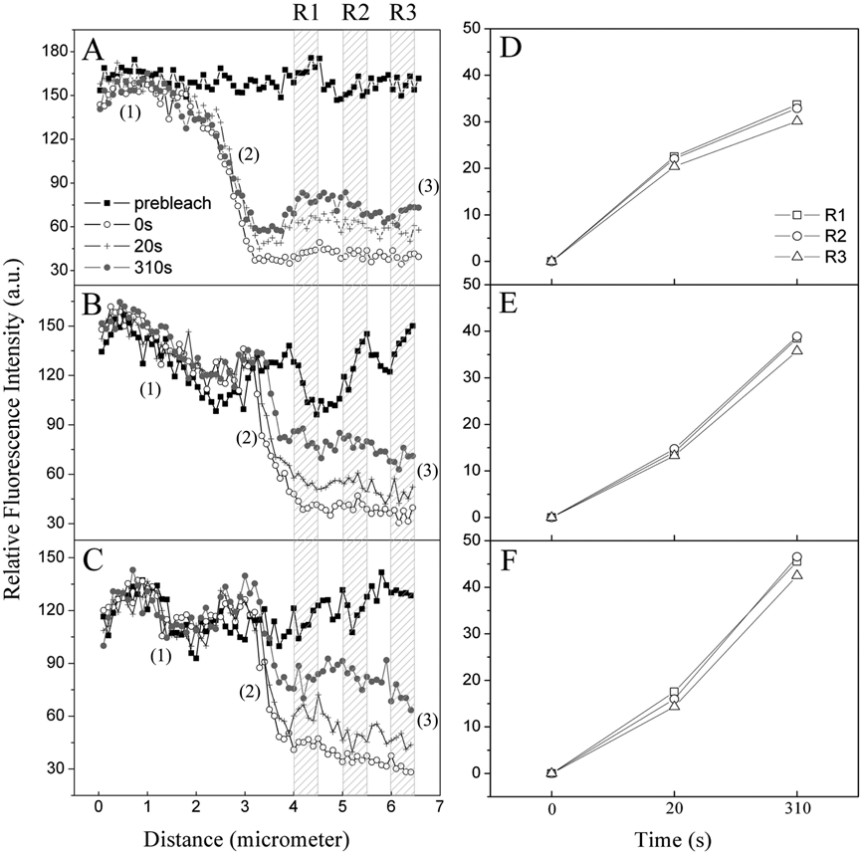
Selected analysis of the fluorescence profiles across the edge of the bleached area in partially-bleached cells, allowing the real-time exploration of the fluorescence dynamics of PBsomes correlated to their lateral diffusion. A, untreated cell; B, betaine-treated cell; C, glutaraldehyde-treated cell. Area (1), the unbleached cellular area; Area (2), the slope of the fluorescence intensity gradient; Area (3), the fluorescence recovery as a function of distance from the edge of the bleached area. The mean fluorescence intensities of three different regions (R1, R2 and R3 in Figure 4A–C) at distinct recovery time were analyzed: D, from untreated cell; E, from betaine-treated cell; F, from glutaraldehyde-treated cell. Fluorescence intensities are normalized at zero second. As shown, no remarkable difference of the fluorescence intensities in these three cellular areas could be observed, indicating the fluorescence recovery of PBsomes does not differ as a function of distance from the edge of the bleached area of theses cells.

Investigations on one-dimensional fluorescence profiles obtained by summing pixel values across the edge of the bleached area (along the X direction in Figure 4) may allow us to analyze the PBsome mobility in real time. As the PBsome complexes diffuse upon the membrane surface, one should observe: (1) a fluorescence loss in the unbleached area if the PBsomes diffuse across the cells (Figure 4A–C); (2) successive flattening of the slope of the fluorescence intensity gradient (Figure 4A–C); (3) changes of the recovery rate as a function of distance from the edge of the bleached area (Figure 4D–F). However, as depicted in Figure 4, the above-mentioned effects were all not obvious, indicating that there was no remarkable difference of the fluorescence behaviors between all native cells, and glutaraldehyde- or betaine-treated cells.

### Fluorescence Recovery of F11 Cells

**Figure 5:**
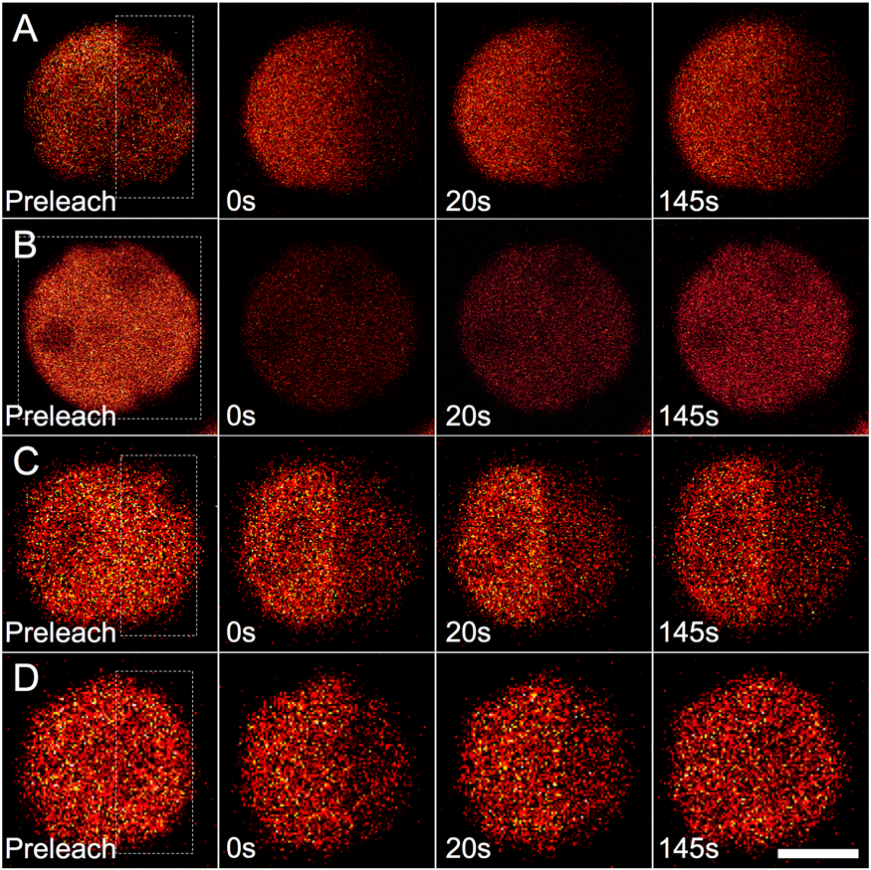
FRAP selected fluorescence images of *P. cruentum* F11 cells. Excitation is at 568 nm and detection range is from 650 to 750 nm. Open squares indicate bleached areas by zooming-in illumination. Scale bar: 5 µm. A, partially-bleached cell; B, wholly-bleached cell; C, partially-bleached cell pretreated with glutaraldehyde; D, partially-bleached cell pretreated with betaine.

*In vitro* single-molecule result has elaborated that two types of PEs in the PBsomes of *P. cruentum* play different roles in the energy transfer [40]. Unlike B-PEs, b-PEs are not involved in the energy decoupling of PBsomes. We measured the cells of *P. cruentum* mutant F11 strain (UTEX 637) which is depleted of B-PEs using FRAP. In Figure 5, the fluorescence recovery of PBsomes in all partially-bleached, wholly-bleached, as well as chemical-fixed cells by glutaraldehyde or betaine was obviously observed, similar to the results of WT cells.

Moreover, PBsomes from the mutant *P. cruentum* F11 cell have a declined dimension due to lower level of PE content [40]–[42]. PBsomes with a smaller size were expected previously to enhance their mobility [31]. However, by examining the FRAP profiles of mutant F11 cell, we found that the fluorescence recovery has similar recovery rates as that of the native cell, suggesting that the fluorescence recovery is not affected by the dimension of PBsomes in red algae.

### Fluorescence Recovery of Ensemble PBsomes *in vitro*

**Figure 6:**
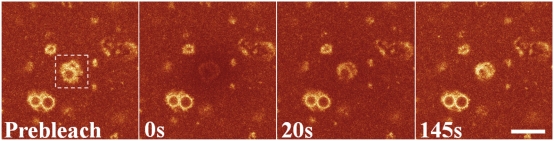
Fluorescence images of isolated PBsomes from *P. cruentum* WT cells. Excitation is at 568 nm and detection range is from 650 to 750 nm. Selected fluorescence images from sequences recorded before bleaching, immediately after bleaching of PBsomes, and at various time lapses. The positions of PBsome aggregations imaged out of the bleaching area did not change, indicating the immobilization of PBsomes above the substrate. Scale bar: 10 µm.

FRAP experiment was also exploited on isolated PBsomes from *P. cruentum*. Intact PBsomes functionally isolated from the thylakoid membrane were spread on the clean surface of glass cover slide. Photobleaching was performed on the pool of immobilized PBsome complexes by zooming-in on a small area of the sample (Figure 6, square). The fluorescence recovery of bleached PBsome aggregations were unexpectedly observed *in vitro* in the first 150 s timespan of our measurements. The fluorescence feature, more specifically the shape of the aggregate, remained fairly stable in the course of the experiment. It revealed that the fluorescence recovery could be detected even in the immobile PBsomes. Taken together, our experimental data strongly demonstrated that the fluorescence recovery observed is most likely ascribed to the intrinsic photoprocesses of the bleached PBsomes *in situ*, rather than the diffusion of PBsomes.

### Fluorescence Properties of PE Emission *in vivo*

In the meanwhile, we studied the fluorescence properties of PE during photobleaching by altering the detecting region from 650–750 nm into 550–600 nm. A lower power of scanning laser (10%) was applied in the prebleaching and postbleaching in order to record continuous fluorescence images, and maximum laser intensity (100%) was introduced to bleach the samples of interest by zooming-in scanning.

**Figure 7:**
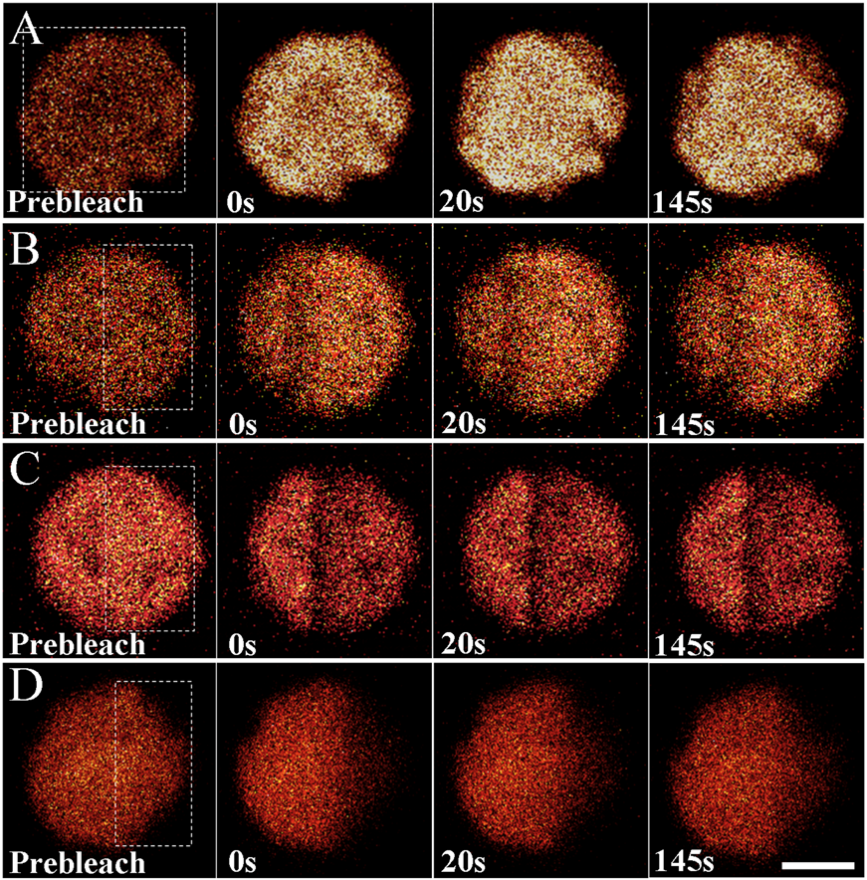
Selected PE fluorescence images in cells imaged with confocal microscopy by detecting fluorescence at 550–600 nm. A, B, native WT cells; C, WT cell pretreated with glutaraldehyde; D, F11 cell. Scale bar: 5 µm.

Figure 7 illustrates selected images of PE fluorescence in the cells in response to intense illumination. Interestingly, wholly (Figure 7A) and partially bleaching (Figure 7B) on the WT cells of *P. cruentum* both resulted in the increase of PE fluorescence intensity. Such increase was also observed in the adjacent non-bleached area near the edges of bleaching region in the partially bleached cells, probably due to the scattered bleaching light. We further studied the PE fluorescent behavior by using glutaraldehyde-treated cell as a control. As a result, the fluorescence increase was not detected (Figure 7C), demonstrating that the increase of PE fluorescence imaged is due to the energetic decoupling of PE molecules within the PBsome rods. Furthermore, experimental results of *P. cruentum* mutant F11 strain, which only contain b-PE assemble with neighboring R-PC [40], did not show the fluorescence increase either (Figure 7D), implying the different roles of B-PE and b-PE in the energetic decoupling. These are corroborated with the previous conclusion obtained from single-molecule experiment [40]. Furthermore, it provides the direct evidence of the energetic decoupling of PBsomes against intense exciting energy *in vivo*. Similar fluorescence increase of PE has also been observed in the FRAP bleach in cryptophyte [43].

**Figure 8:**
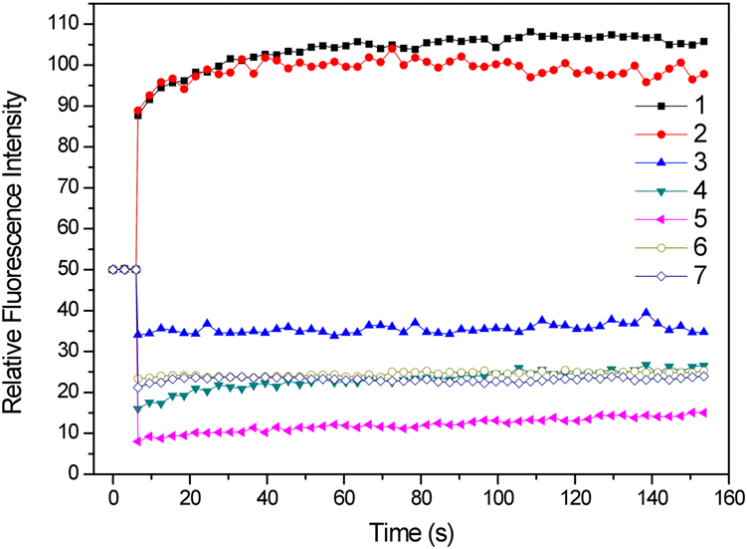
Time-lapse PE fluorescence intensity in cells in the process of photobleaching. Powerful irradiation can cause initial increase of PE and prolonged illumination time may induce bleaching of PE. (1), whole bleaching of native WT cell; (2), 5 times scan; (3), 10 times scan; (4), 20 times scan; (5), 30 times scan; (6), WT cell pretreated with glutaraldehyde; (7), F11 mutant cell.

Figure 8 shows quantitatively the fluorescence intensities of PE in cells as a function of time. It is seen that the bleaching on WT natural cell induced the increase of PE emission (curves 1 and 2), and longer irradiation time of photobleaching was capable to trigger more remarkable fluorescence reduction (curves 2, 3, 4 and 5). The PE fluorescence intensity of the bleached areas in WT cells pretreated with glutaraldehyde and F11 cells is relatively constant, lower than the initial fluorescence intensities before photobleaching (curves 6 and 7). The plots revealed that there is no significant recovery of PE, either from increased or declined fluorescence levels. This suggested, on the other hand, that there is no significant mobility of PEs that are the major components of PBsomes.

### Fluorescence Recovery of PEs in Ensemble PBsomes *in vitro*

**Figure 9:**
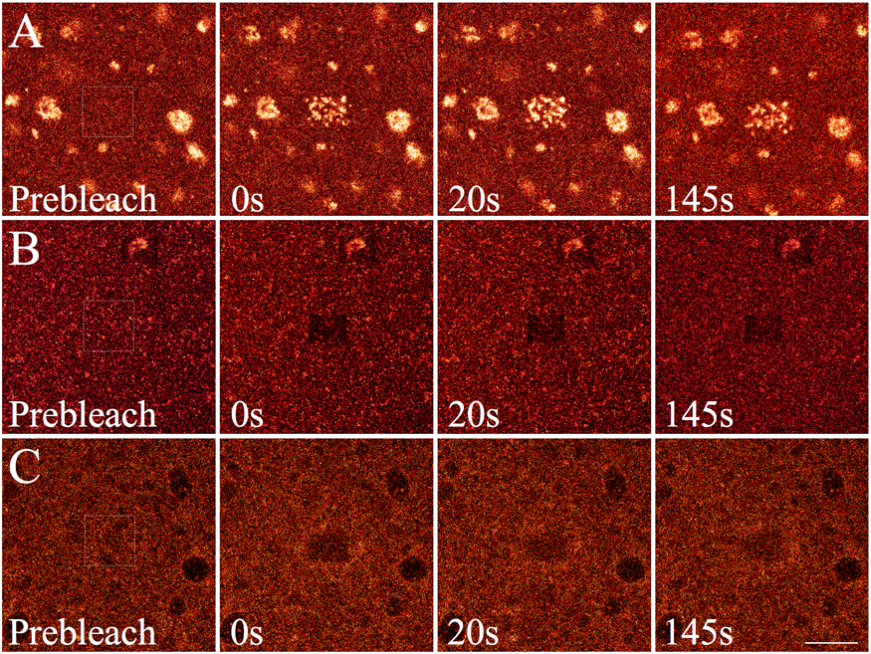
Selected PE fluorescence images of ensemble PBsome isolated from *P. cruentum* WT cells (A), WT cell pretreated with glutaraldehyde (B) and F11 mutant (C) obtained with confocal microscopy by detecting 550–600 nm. Scale bar: 5 µm.

Figure 9 shows the fluorescence dynamics of PE in isolated PBsomes upon intense illumination at 514 nm. Immediately after photobleaching, continuous scan recorded a dramatic fluorescence increase in the wholly-bleached area of WT PBsomes (Figure 9A), whereas PE fluorescence was bleached in both WT PBsomes treated with glutaraldehyde (Figure 9B) and B-PE-lacking PBsomes from the F11 mutant (Figure 9C). This further confirmed the energetic decoupling of PBsomes in response to intense exciting light.

## Discussion

Thin-section electron microscopic images of *P. cruentum* cell present a globular shape with a typical diameter of 5–8 µm [44], [45]. The large chloroplasts consist of parallel-arranged and unstacked lamellae that occupy most of the intra-cellular space *in vivo*. Such a regular thylakoid organization is supposedly suitable for FRAP measurements [21], [22].

In cyanobacteria, PBsomes have been suggested to diffuse rapidly on the surface of thylakoid membrane [20]. However, recent investigations have shown that the thylakoid membrane in the unicellular red alga *P. cruentum* exhibits dense crowding of PBsome complexes [27], [28]. In addition to determining the arrangements of PBsomes on the thylakoid membrane, the supramolecular crowding has also profound implications for the dynamic behavior of PBsomes in *P. cruentum*: the rapid and long-range movement of PBsomes may be inhibited by the dense packing of membrane surface, and the limited free vertical spacing between opposite thylakoid layers, as well as remarkable steric hindrance, taking into account the large size of individual PBsomes. Furthermore, PBsomes attach quite strongly to the membrane surfaces [46], whereas the rapid diffusion of PBsomes requires looser association of PBsomes to the photosynthetic reactions centers.

This contradiction was explored in the present work by studying the fluorescence dynamics of PBsomes on thylakoid membranes from *P. cruentum* using FRAP. First, FRAP results of WT cells present partial fluorescence recovery. Assuming the fluorescence recovery is attributed to the diffusion of PBsome complexes [20], it then indicated that some PBsomes are presumably immobile on the thylakoid membranes of red algae. However, this probability was not supported by the finding that the fluorescence recovery of PBsomes declines as the illumination strength increases. The partial fluorescence recovery was also observed in partially-bleached F11 cells, wholly-bleached WT and F11 cells, and even in immobilized PBsome complexes *in vitro*. We also detected, similar fluorescence recovery behavior of *P. cruentum* cells pretreated with protein cross-linking agents glutaraldehyde or betaine with the fixation effect. By comparative analysis of the fluorescence feature and recovery rate, no significant difference of the fluorescence behavior between those cells was observed. All these data strongly indicate the observed recovery is not likely ascribed to the lateral diffusion of PBsome complexes on the thylakoid membranes.

Another evidence of the PBsome diffusion in red algae was provided by recording the PE fluorescent signal instead of the fluorescence emission of PBsome terminal emitters. After photobleaching, rapid diffusion of PBsomes was expected to cause the recovery of PE fluorescence to the initial fluorescence intensity. Alternatively, we could not observe such tendency in either the area of WT cells having increased PE fluorescence intensity or the area of glutaraldehyde/betaine-pretreated WT cells and F11 cells showing decreased PE fluorescence intensity (Figures 7 and 8).

Based on all the observations we conclude that, lateral diffusion of red algal PBsomes is not the predominant mechanism which could potentially explain the fluorescence recovery that we observed by means of FRAP. Instead, the fluorescence recovery is probably ascribed to the intrinsic photoprocesses of the bleached PBsomes *in situ*, rather than the rapid diffusion of PBsomes on thylakoid *in vivo*, although the mechanism of the intrinsic photoprocesses still remains to be determined.

The mechanism for light-state transition in red algae remains controversial. Unlike the state transition in higher plant, light-dependent phosphorylation was absent in cyanobacteria and rhodophytes [47]. Rapid mobility of PBsomes in cyanobacteria has been proposed to be required for the PBsome-dependent state transition [31]. In red alga *P. cruentum*, the long-distance diffusion of PBsome complexes may be highly restricted, however potential diffusion of PBsomes confined to small domains at sub-optical scales would not be excluded. For instance the local conformational changes and movement of PBsomes between adjacent photosystem II and photosystem I complexes [48] can probably be involved in the energy redistribution in red algae.

By monitoring the fluorescence of PEs in the PBsomes, we studied the fluorescence dynamics of ensemble PBsomes *in situ* during photobleaching. We demonstrated directly the energetic decoupling of PBsomes *in vivo* in response to the bleaching laser. The PEs (specifically B-PEs), rather than PC/APC, are mainly involved in the energetic decoupling. This is in agreement with single-molecule observations [40] and ensemble results of isolated PBsomes (data not shown). Such an energetic decoupling of PBsomes is proposed to have important implication on the photoprotective role of PBsomes: It may allow excess photon energy from PE to photosynthetic reaction centers to be modulated to minimize the risk of chlorophyll photooxidation. Therefore, like the light-state transition, this mechanism might contribute to prevent PE-containing algae from excessive photoinhibition.

Photosynthetic membrane has been considered as an ideal model to study the protein mobility in view of their naturally fluorescent properties [21]. However, the complexity of photosynthetic membrane should be taken into account in FRAP, as numbers of fluorescent protein are located densely in the membrane with a close functional association benefiting for efficient energy transfer. Not only does the spectral overlap make it hard to distinguish the individual contribution of each complex [20], but also the photoreactions among distinct cooperated protein complexes can be triggered by exposure laser, for instance the energetic decoupling in the PBsomes as we observed and the photoblinking behaviors revealed in single-molecule studies on phycobiliproteins [49]–[52]. In addition, it was proposed that all the fluorescent molecules can undergo reversible photobleaching [53]. Therefore, investigations on each functional protein complex involved may be prerequisite for understanding the fluorescence dynamics of photosynthetic membrane.

## Materials and Methods

### Growth of Cells and Separation of Intact PBsomes

*P. cruentum* WT and mutant F11 strain UTEX 637 were grown in an artificial sea water medium [54]. Flasks were supplied with 3% CO_2_ in air through a plug of sterile cotton at a constant temperature of 20°C. Cultures were illuminated continuously with light provided by daylight fluorescent lamps at 6 W·m^−2^. Intact PBsomes were separated following the previous protocol [27].

### FRAP Measurements

Growing cells or cells with corresponding pre-treatments by 1% (v/v) glutaraldehyde or 0.5 M betaine buffer were immobilized on a slide covered with a glass slip and located under objective lens of laser scanning confocal fluorescence microscopy (LSCM).

FRAP experiments were carried out with a Leica TCS NT confocal microscope. Samples were excited with a 568 nm Yellow Krypton laser (10 mW) or a 514 nm Argon laser (10 mW). PBsome emission was detected at 650–750 nm or 550–600 nm for detecting PBsome emission and PE emission, respectively. After recording a prebleaching image, the sample was bleached by zooming in and scanning with maximum laser power for three seconds. Subsequently, images were captured with the same power as that of pre-bleaching image. No detectable photobleaching during the recording of successive image scans was observed with optimized laser power.

The fluorescence recovery curve is fitted by single exponential function, given by
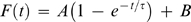
(1)where *F(t)* is the intensity as time *t*; *A* and *B* are the amplitudes of the time-dependent and time-independent terms, respectively; *τ* is the lifetime of the exponential term (time constant), and the recovery rate is given by *R = 1/τ*.
